# The Safety of Using Body-Transmit MRI in Patients with Implanted Deep Brain Stimulation Devices

**DOI:** 10.1371/journal.pone.0129077

**Published:** 2015-06-10

**Authors:** Joshua Kahan, Anastasia Papadaki, Mark White, Laura Mancini, Tarek Yousry, Ludvic Zrinzo, Patricia Limousin, Marwan Hariz, Tom Foltynie, John Thornton

**Affiliations:** 1 Sobell Department of Motor Neuroscience & Movement Disorders, UCL Institute of Neurology, London, United Kingdom; 2 Lysholm Department of Neuroradiology, National Hospital for Neurology and Neurosurgery, UCLH NHS Foundation Trust, London, United Kingdom; 3 Department of Brain Repair and Rehabilitation, UCL Institute of Neurology, London, United Kingdom; University of Minnesota, UNITED STATES

## Abstract

**Background:**

Deep brain stimulation (DBS) is an established treatment for patients with movement disorders. Patients receiving chronic DBS provide a unique opportunity to explore the underlying mechanisms of DBS using functional MRI. It has been shown that the main safety concern with MRI in these patients is heating at the electrode tips – which can be minimised with strict adherence to a supervised acquisition protocol using a head-transmit/receive coil at 1.5T. MRI using the body-transmit coil with a multi-channel receive head coil has a number of potential advantages including an improved signal-to-noise ratio.

**Study outline:**

We compared the safety of cranial MRI in an *in vitro* model of bilateral DBS using both head-transmit and body-transmit coils. We performed fibre-optic thermometry at a Medtronic ActivaPC device and Medtronic 3389 electrodes during turbo-spin echo (TSE) MRI using both coil arrangements at 1.5T and 3T, in addition to gradient-echo echo-planar fMRI exposure at 1.5T. Finally, we investigated the effect of transmit-coil choice on DBS stimulus delivery during MRI.

**Results:**

Temperature increases were consistently largest at the electrode tips. Changing from head- to body-transmit coil significantly increased the electrode temperature elevation during TSE scans with scanner-reported head SAR 0.2W/kg from 0.45°C to 0.79°C (p<0.001) at 1.5T, and from 1.25°C to 1.44°C (p<0.001) at 3T. The position of the phantom relative to the body coil significantly impacted on electrode heating at 1.5T; however, the greatest heating observed in any position tested remained <1°C at this field strength.

**Conclusions:**

We conclude that (1) with our specific hardware and SAR-limited protocol, body-transmit cranial MRI at 1.5T does not produce heating exceeding international guidelines, even in cases of poorly positioned patients, (2) cranial MRI at 3T can readily produce heating exceeding international guidelines, (3) patients with ActivaPC Medtronic systems are safe to be recruited to future fMRI experiments performed under the specific conditions defined by our protocol, with no likelihood of confound by inappropriate stimulus delivery.

## Introduction

Deep brain stimulation (DBS) is an established treatment for patients with advanced Parkinson’s disease (PD) [1,2] and is being explored in a growing list of other neurological and psychiatric diseases [3,4]. DBS delivers chronic electrical stimulation to deep brain structures via implanted multi-electrode leads and an implantable pulse generator (IPG) implanted either in the pectoral area or abdominal wall.

Functional neuroimaging permits whole brain *in vivo* assessment of neural function and has previously been used to explore the pathophysiology of PD [5–7], and the effect of DBS in PD patients [8–10]. The majority of the literature has used positron emission tomography or single-photon emission computed tomography to map brain regions that are modulated by DBS [11–17]. A handful of functional MRI (fMRI) studies have been undertaken, however, most have taken place immediately after electrode implantation [18,19]. The increased spatial and temporal resolutions afforded by fMRI, as well as the enhanced modelling techniques available [20], suggest fMRI could provide important insights into the neuromodulatory effects of DBS. This may help clarify the underlying mechanisms of DBS, allowing efficacy improvement and translation to other disorders. Thus, the safety of MRI in patients with fully implanted DBS systems is an important consideration.

The paucity of fMRI studies in DBS patients has been largely due to concerns regarding the safety of scanning patients with implanted systems [21]. Two notable and unfortunate case studies have highlighted the potential dangers of DBS interacting with MRI scanning when safe operating conditions are not observed [22,23]. Thus it is important to establish MRI protocols that both minimise risk to the patient, while maximising the quality of the resulting clinical or research data. To date, safety considerations have led to some compromise in the quality of fMRI data attainable with DBS apparatus *in situ*, particularly the specification of a head-only RF transmit coil effectively precluding the image-quality advantages of multi-channel receive coil technology [24,25]. Furthermore, the contemporary gold standard for fMRI in the cognitive neuroscience literature is to use body-transmit MR with multi-array receive coils (usually 16 or 32 channels), thus we aimed to bring DBS fMRI in line with best practice. This work assesses the safety of MRI in an *in vitro* model of a DBS patient using a body RF transmit coil, in comparison to identical acquisitions using a head-transmit coil in accordance with our established clinical and research practice. We then explore potential technical confounds to fMRI studies using the body-transmit coil. However, before reporting our safety experiments, we now briefly review the theory of potential interactions between implanted DBS equipment and the electromagnetic environment in MRI.

### DBS MRI interactions: Theory

**Fig 1:**
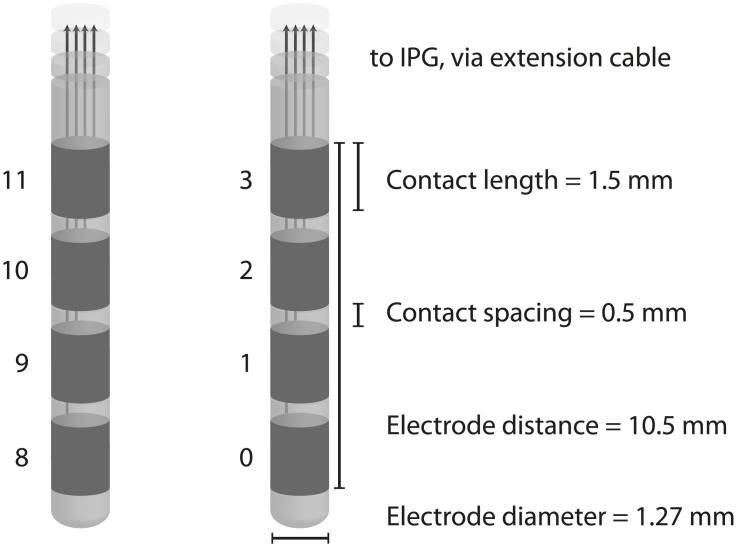
The Medtronic 3389 deep brain stimulation electrode studied in this work. The deepest (most distal) contacts are 0 and 8.

The DBS system comprises at least three implanted components; (1) the electrodes (‘leads’), (2) an implantable pulse generator (IPG) or ‘pacemaker’, and (3) extension cables connecting the IPG to the electrodes. All components contain some metallic materials including platinum-iridium, stainless steel, titanium and silver as part of the conducting circuit or casing. Four metal contacts lie at the end of each electrode (Fig 1), in contact with the target neural tissue, and a voltage is induced either between a contact and the IPG case (monopolar stimulation) or between two adjacent contacts (bipolar stimulation), causing current to flow through the target tissue.

The major, well documented MRI safety concern is rapid temperature increases at the tips of the electrode contacts due to coupling of the electrical component of radiofrequency (RF) oscillating electromagnetic field excitation pulses applied during MRI to the DBS circuit (known as ‘resonant coupling’ or ‘the antenna effect’) [18,19,26–31]. The resulting induced currents produce heating, elevating tissue temperature to potentially dangerous levels. Furthermore, there is an additional risk that voltages induced in the DBS circuit during MRI could lead to potentially harmful uncontrolled neural stimulation independent of IPG function.

### Implications for functional MRI studies

Assuming safe operation can be established, gradient echo echo-planar imaging (GE-EPI) fMRI comparing blood oxygenation level dependent (BOLD) signal ‘ON’ and ‘OFF’ DBS presents an important avenue of research. However, there are a number of potential experimental confounds. Most fundamentally, the simplest study design would assume that the IPG output is not disrupted by the MRI environment and therefore it is essential that the IPG accurately delivers the intended stimulation pulse trains during fMRI, otherwise the states compared would not relate to the true DBS ‘ON’ and ‘OFF’ conditions. In addition, MRI-induced tissue temperature changes could theoretically also confound experimental data physiologically [32–36] potentially altering the assumed constant haemodynamic coupling between blood flow and neural activity. Thirdly, the MRI proton resonant frequency is sensitive to tissue temperature thus local RF-induced temperature increases could cause confounding signal changes during the fMRI experiment independent of activation.

### Evidence for MRI-induced heating

Previous *in vitro* results using various media modelling the thermal and electrical characteristics of neural tissue suggest that electrode heating during MRI depends on factors including coiling of the DBS leads [37], IPG type and brand [28], MRI sequence specific absorption rate (SAR) [31], and field strength [38], as well as the geometry of the RF transmit coil relative to the leads [21]. We have previously shown that heating in a Medtronic Kinetra DBS system (Medtronic Inc., Minneapolis, MN, USA) remains under 1°C if scanning is restricted to 1.5T, using a transmit-receive head coil, and limiting scanner-reported sequence head SAR to less than 0.4W/Kg [27]. Furthermore, we showed that common fMRI sequences did not interfere with IPG stimulus delivery in our arrangement, and thus would not confound ‘ON’ vs. ‘OFF’ DBS stimulation condition studies. Under these restrictions, at our centre we routinely perform post-implantation electrode placement verification MRI as part of our clinical practice [39]. We have additionally recently completed fMRI studies in patients with fully implanted DBS systems undergoing chronic stimulation without safety incident, providing new insights into the neuromodulatory effects on cortico-subcortical connectivity [9,10].

The established use at our centre and elsewhere of a head-transmit/receive coil is thought to maximise safety by minimizing the area of the DBS circuit exposed to the MRI RF pulses. However, in receive mode, such coils typically exhibit a lesser filling-factor than equivalent receive-only coils commonly available, reducing the available signal-to-noise ratio (SNR). This approach also precludes the use of multi-array head receive coils that confer additional SNR and image quality advantages. As well as mitigating these disadvantages, use of the body-transmit coil mode could theoretically allow patients with *in situ* DBS access to MRI for radiological indications requiring investigations other than of the head, e.g. MRI spine, abdominal or limb examinations. That being said, this work only addresses cranial MRI.

Additionally, since MRI safety is dependent on the specific DBS system manufacturer-type and model, it is important to confirm the MRI safety of new DBS systems introduced subsequently to previously published safety assessments.

### Aims

The aims of this study were, through *in vitro* testing, to:

1. Determine safe conditions for head-transmit/receive coil MRI with current state-of-the-art DBS hardware.
2. Compare the MRI-induced heating obtained using the body-transmit coil to that with the head-transmit coil under both 1.5T and 3T field strengths.
3. Compare the MRI-induced heating between DBS ‘ON’ and ‘OFF’ stimulation conditions.
4. Confirm that fMRI sequences using the body-transmit coil do not impair IPG function.

## Materials and Methods

Standard radiological orientation is used throughout this report. A poly-methyl-methacrylate phantom with dimensions resembling a human torso was filled to a depth of 10cm with a gel of poly-acrylic acid partial sodium salt (8 g/L), sodium chloride (0.70 g/L), and distilled water [27,40]. At room temperature, this gel has been demonstrated to possess electrical and thermal characteristics similar to those of human tissue [27,41].

**Fig 2:**
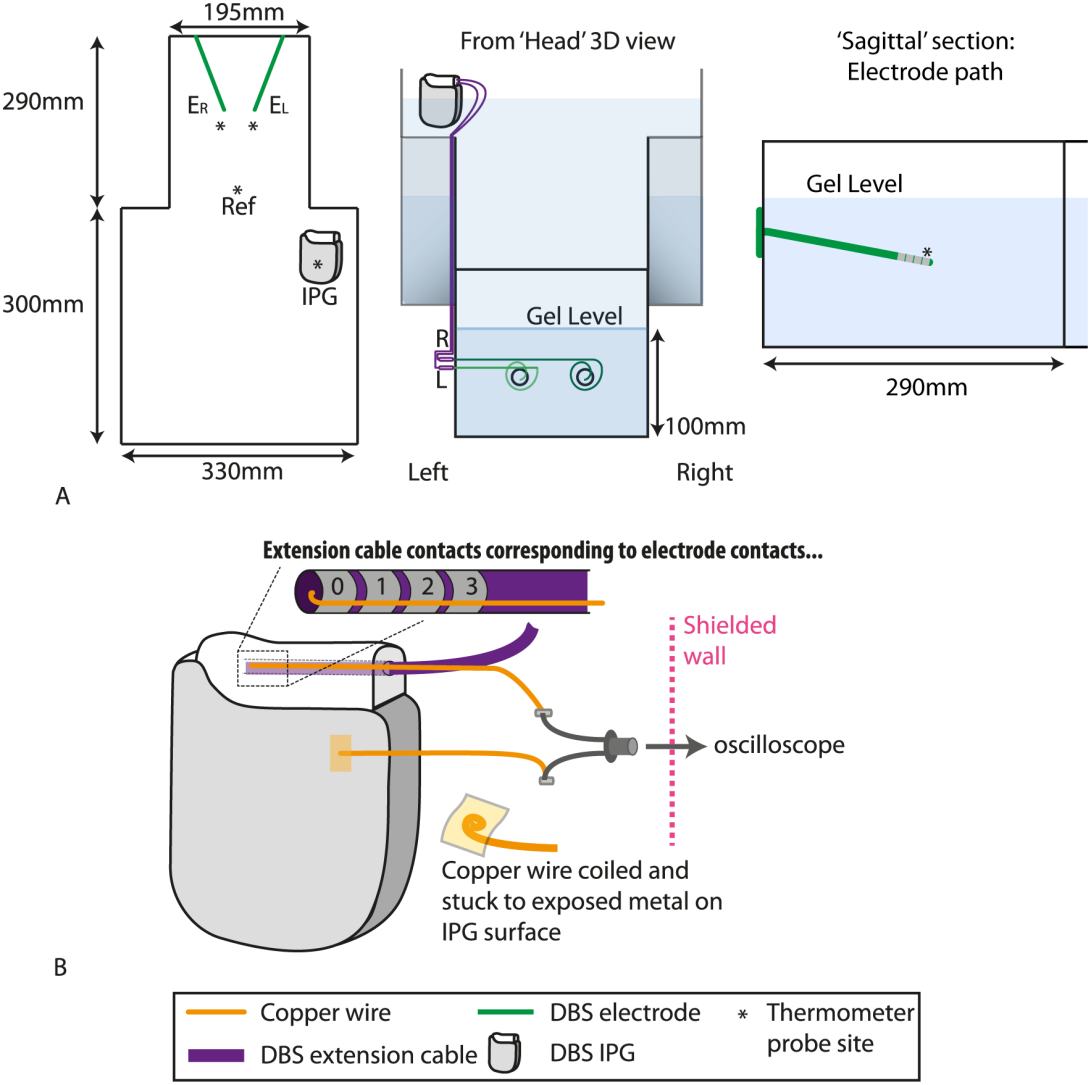
In vitro model of DBS patient implanted at this centre. Note that the extension cables are routed along the *external* surface of the phantom. (A) Phantom dimensions. * = thermometer recording site, IPG = Implantable pulse generator. (B) Recording the voltage output of the IPG. EL = left electrode, ER = right electrode.

A Medtronic ActivaPC DBS system (Medtronic Inc, Minneapolis, MN, USA) was positioned within the phantom in a configuration resembling that in a patient with fully implanted hardware. The IPG (Model 37601) was partially submerged in the left ‘pectoral’ region of the phantom such that the outer casing was in contact with the gel. Two 18mm diameter burrholes were drilled into the superior edge of the phantom, representing the superior cranium, and burr-hole caps were fixed into position (Fig 2).

The simulated target nuclei positions were established at fixed locations analogous to the positions of the subthalamic nuclei (STN) relative to the burr-holes in a human (based on measurements from STN-DBS implantation surgeries). Two electrodes (model 3389, Medtronic Inc, Minneapolis, MN, USA), one on each side, were fixed to the burrhole caps, and their ends were positioned in the target ‘nuclei’ regions. Suture silk and a plastic frame were used to maintain the path of the electrode leads through the gel. Excess lead was coiled anti-clockwise around the burrhole 1–3 times on the external surface of the phantom, in an arrangement similar to our clinical practice. The leads were connected to the IPG using two DBS extension cables (Model 37085) routed along the external surface of the phantom.

### MRI scanning

Measurements were performed in Siemens Magnetom Avanto (1.5T) and TIM Trio (3T) MRI systems, both operating at Siemens software level VB17. The phantom was placed head first, supine within the bore of the scanner, with the tips of the electrodes at the magnet isocentre (i.e. using the tips of the electrodes as a central landmark for image-volume prescription). The scanner calculated predicted SAR values assuming the phantom was a 75 kg, 44 year old male. Imaging was performed with either the respective manufacturer-supplied head transmit-receive coil, or using the system body-transmit coil with a 12-channel head receive coil. To provide temperature changes of sufficient magnitude to assess reliably while minimizing the risk of damaging the DBS equipment, turbo-spin echo (TSE) sequences (repetition time (TR) 4000ms; echo time (TE) 111ms; refocussing flip angle (FA) 120°; excitation FA 90°, bandwidth (BW) 100Hz/pixel; field of view (FOV) 22x25cm; matrix 320x320; echo-train length 12, 4 slices; slice thickness (ST) 2mm; 4 averages; scan time 6 minutes 14 seconds) with scanner-reported head SAR 0.2W/kg (body SAR ≈ 0.1 W/Kg) were used as a medium SAR exemplar sequence that would not ordinarily be performed on a patient with implanted equipment, but produced sufficient heating to act as a positive control for our thermometry system. Measurements were performed twice for each coil combination used, and twice for each DBS stimulation condition, with times between scanning sessions minimised to avoid changes in gel properties, e.g. due to evaporation. The head SAR calculated by the scanner was recorded for each scan.

For each MRI sequence investigated, temperatures were also recorded during the ‘pre-scan’ calibration procedures (RF power calibration and B_0_ field homogeneity optimisation) performed automatically by the scanner prior to the main sequence execution.

Additionally, at 1.5T we examined the effect of changing the phantom position relative to the body-transmit coil upon electrode heating, in this case with the IPG set to ‘OFF’ for all measurements. These measurements were performed to explore the safety implications of cranial MRI with patients misplaced in the scanner, i.e. positioned with the electrode tips away from the scanner magnet isocentre. The phantom was first positioned with the tips of the electrodes at magnet isocentre and then displaced 150mm *into* (negative displacement) the scanner, and thermometry performed during the TSE acquisition. The measurement was then repeated at six further successive displacements relative to the magnet isocentre (-100mm, -50mm, 0mm, +50mm, +100mm, and +150mm).

To directly confirm the safety of a typical research fMRI acquisition compared to our positive control TSE acquisition protocol, we performed additional 1.5T thermometry with the phantom positioned with the electrode tips at the magnet isocentre during a GE-EPI acquisition (TR 3700ms; TE 40ms; FA 120°; BW 2298Hz/pixel; FOV 19.2cm; matrix 64x64; 49 slices; ST 2.5mm; 96 measurements 1 average; scan time 6 minutes). The effect of phantom position within the scanner was not explored with this sequence.

### IPG settings

Scanning was performed with the IPG active (‘ON’) and inactive (‘OFF’). During ON scans, the IPG was programmed to deliver stimulation typical of that employed in therapeutic STN DBS for PD (Frequency: 130Hz; Amplitude: 3.5V; Pulse width: 60μs, bilaterally). Unipolar stimulation settings were used so that current flowed from the case to the distal (the deepest) contacts of each electrode (RHS = contact 0, LHS = contact 8).

### Fibre-optic thermometry

**Fig 3:**
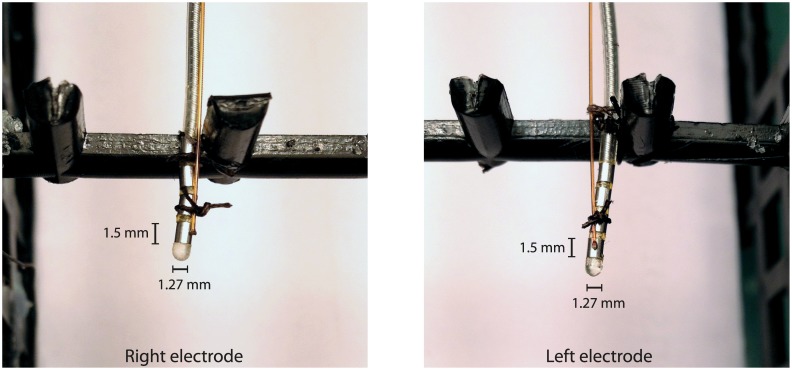
The fibre-optic temperature sensors positioned at the distal electrode contacts. The fine golden leads are the temperature probe optical fibres, whereas the grey leads with 4 visible metal contacts are the DBS electrode leads. Suture silk was used to ensure that the thermometer probes were in close thermal contact with the distal electrode contact surfaces, previously demonstrated to be the sites of greatest MRI-induced heating.

The temperature was recorded simultaneously from four loci in the phantom using a 4 channel fibre-optic temperature thermometer (Neoptix ReFlex—Neoptix, Québec, Canada) based on gallium arsenide (GaAs) semiconductor crystal technology (sampling rate = 1Hz). Temperature probes were located at the distal electrode contacts (one for each electrode lead—see Figs 2 and 3), the IPG case, and the centre of the phantom ‘head’ region, remote from the electrode contacts, this location providing a control recording of background temperature changes.

### IPG voltage output in during typical fMRI sequences

To confirm that the electromagnetic fields played out by the scanner during fMRI sequences did not disturb IPG function, in a separate experiment the voltage between contact 0 (i.e., the most distal contact on the quadripolar lead) and the IPG case was measured whilst the phantom was exposed to the representative fMRI sequence. Fine enamelled copper wire (Diameter: 0.15mm; Gauge: 40swg) was threaded into the extension cable socket in the IPG, such that the bared-end of the wire connected with the IPG distal electrode contact. A separate wire was in electrical contact with the IPG case and the wires were connected to a gold-plated and nickel-plated brass oscilloscope probe that was firmly secured to the external wall of the phantom. This was in turn connected via a coaxial extension cable to a digitizing oscilloscope (WaveJet 354A, Teledyne LeCroy—Bandwidth = 500 MHz) (Fig 2b) in the MRI control room. The voltage was recorded both with and without an active IPG output before scanning sessions to confirm the captured signals accorded with the voltage and frequency that the IPG had been programmed to deliver. Temperature data were not collected during these scans as the additional wiring involved may have confounded heating estimates.

### Effect of lead immersion in phantom gel

For experimental simplicity and consistency with our previous measurements [27], for the above measurements the DBS leads and extension cables were routed along the *outside wall* of the phantom. This meant that this part of the DBS circuit was only partly immersed in the phantom gel. Since the electrical properties of the medium around the leads may in principle influence lead heating [42], we performed a simple test to investigate the impact of this upon our *in vitro* model: The apparatus was reassembled with the lead extensions and excess-lead coiling fixed to the *inside* of the phantom walls, immersed in the gel. At 1.5 Tesla, the phantom was exposed to the same TSE 0.2W/Kg sequence, and the electrode tip temperature recorded as previously.

Then, leaving the excess-lead coil and electrodes positions within the gel unchanged, the lead extensions were re-routed along the outside wall of the phantom and the measurement repeated. To assess reproducibility these measurements were repeated in 2 sessions separated by 1 week.

### Data Analysis

#### Maximum heating estimates

Data was analysed using Matlab (The MathWorks, Inc., Natick, MA, USA). Fifteen pre-scan temperature measurements from each probe were averaged to provide a probe-specific baseline temperature. Temperature change (ΔT) was calculated by subtraction of this baseline value from each time-point during the scan and for up to 1 minute post-scan to allow time for the temperature to stabilise. To remove occasional instrumentally-generated noise, data were thresholded to remove implausibly extreme values (mode +/- 80°C), and values associated with implausibly large temperature changes between data points, i.e. where the first differential w.r.t. time was greater than > 1.9°C/sec. These processing criteria were optimised using pilot data collected in the 3T scanner with the body-transmit coil displaying the greatest and fastest increases in temperatures, and were then applied identically to all measurements.

Pilot thermometry measurements demonstrated a characteristic exponential increase in temperature at the electrode tips, the rate of increase decreasing as the scan progressed, with the most extreme heating occurring at the left electrode tip, consistent with our previous findings with a similar arrangement [27]. In light of this time-course, a 20 second epoch was extracted at the end of each scan (i.e. during the plateau phase, whilst the electrode was at its highest temperature) to represent the maximum temperature in each case. Thus there were 20 data points for each MRI scan; each scan sequence was tested twice, thus there were 40 data points per coil combination per stimulation condition. Using the left electrode data as the worst-case **Δ**T estimate, paired T tests were used to compare MRI-induced **Δ**T produced by the head-transmit and the body-transmit coils respectively (collapsing across stimulation conditions), and similarly between stimulation conditions (collapsing across coil conditions).

#### The effect of test-object position relative to the body-transmit coil

Linear regression analysis was used to determine if the position of the phantom predicted the observed heating effect. The electrode tip position at the isocentre was defined as 0cm; displacement *into* the scanner (i.e., equivalent to *pushing* a subject’s feet further into the scanner) was coded as a negative displacement, whereas displacement *out of* the scanner (i.e., equivalent to *pulling* a subject’s feet out of the scanner) was coded as positive displacement. Mean ΔT at the left electrode tip during the final 20 second epoch at each position was considered the dependent variable, with position the independent variable.

#### IPG output during MRI scanning

Voltage waveforms before and during a GE-EPI fMRI sequence were captured with the digital storage oscilloscope and transferred electronically into MATLAB for plotting and analysis. The DBS pulse period was calculated by averaging the time between voltage excursions greater than 2.5V. The DBS frequency was calculated as the inverse of the period, i.e. *f = 1 / T*.

## Results

**Fig 4:**
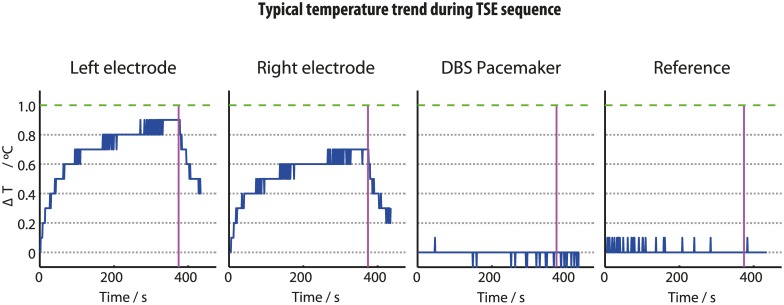
Typical temperature time courses observed during a TSE sequence at the 4 thermometer sites using the body-transmit coil. Green line is our safety threshold (1°C). Pink line indicates the end of the scan. Note the TSE sequence acted as positive control, producing temperature increases greater than the sequences used for subjects with in situ DBS at our centre.

Baseline temperatures before the measurements commenced were between 18–20°C. At the electrode tips, the TSE sequences produced a **Δ**T of <1°C and <2°C at 1.5T and 3T respectively, regardless of transmit coil choice. Any temperature increases at the IPG body were <0.2°C in all cases. The reference probe confirmed that gel temperature changes distant from any DBS hardware remained within the range of the thermometer sensitivity (±0.1°C). In accordance with our previous results [27], the left electrode consistently displayed greater heating than the right. Regardless of coil or stimulation setting, electrode contact **Δ**T followed a similar time course (Fig 4); an initial rapid increase in temperature lasting approximately 50 seconds, followed an exponential recovery eventually tending towards a plateau at a maximum temperature at the end of the MRI pulse sequence. When the scan ended, temperatures rapidly returned towards baseline.

As a representative assessment of measurement reproducibility and gel stability we include in the supplementary material data showing no significant difference between otherwise identical measurements performed at 1.5T, 8 days apart. Exposure to MRI at both 1.5T and 3T had no effect on the IPG’s ability to turn on/off and communicate with the DBS patient controller. With the phantom positioned at the magnet isocentre, the scanner-reported head SAR remained at 0.2 W/Kg for the prescribed TSE sequence (body SAR ≈ 0.1 W/Kg), and was 0.1 W/Kg for the GE-EPI sequence, regardless of coil used. (Note: reported SAR levels are quantized for display purposes in divisions of 0.1W/Kg, so the GE-EPI sequence in fact delivered 0.064W/Kg).

### Pre-scan induced heating

**Fig 5:**
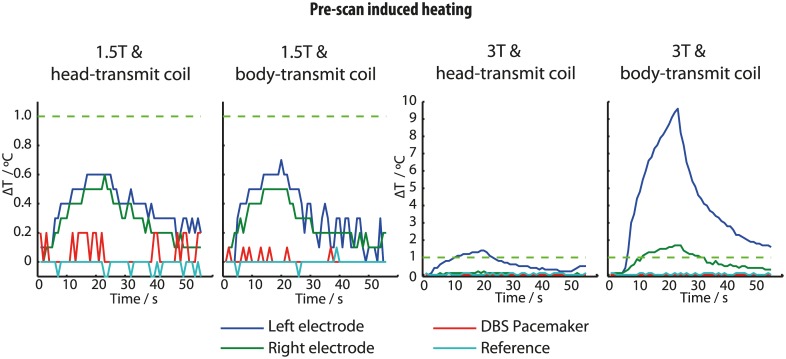
Temperature increases at the 4 measurement sites during ‘pre-scan’ system calibration periods. Note the change in scale for the 3T plots.

Prior to imaging acquisitions that followed changes in phantom position, the scanners automatically performed a ‘pre-scan’ calibration procedure lasting approximately 30 seconds. Scanner-induced **Δ**T were also observed during this pre-scan period, specifically during the 3D shim-field estimation. At 1.5T, pre-scan **Δ**T were similar to the maximum values observed during the TSE image-data acquisitions, regardless of coil used. At 3T, pre-scan **Δ**T using the head-transmit coil were similar to the TSE scan heating. However using the body-transmit coil at 3T, **Δ**T at the left electrode approached 10°C during the pre-scan procedure, far exceeding **Δ**T during the main acquisitions (Fig 5).

### Effect of body-transmit coil on electrode heating

**Fig 6:**
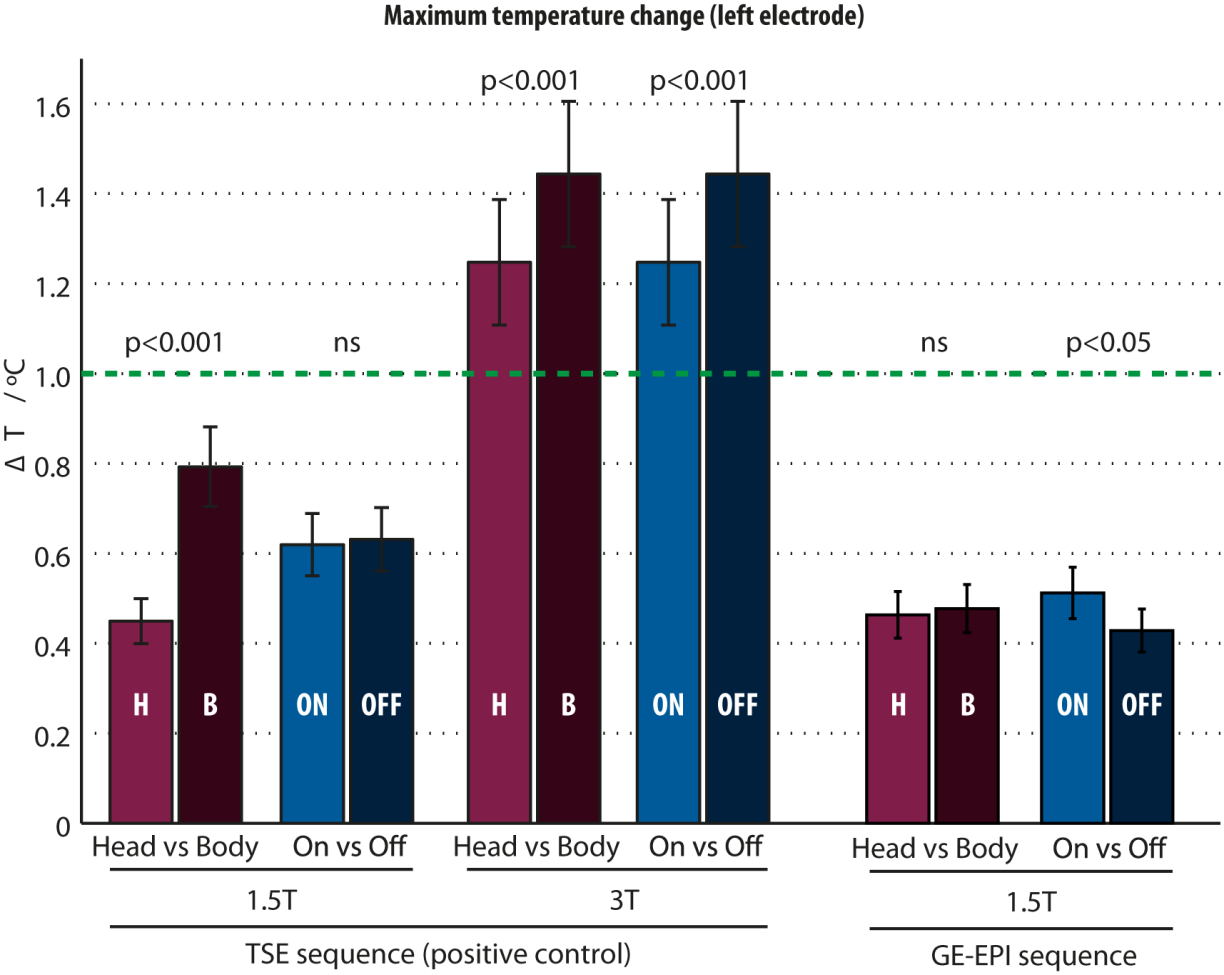
Maximum electrode-tip temperature change induced by the TSE sequence at 1.5T and 3T systems, and GE-EPI sequence at 1.5T.

Changing from head- to body-transmit coil produced small but significant increases in the observed electrode tip maximum **Δ**T during TSE scans, from a mean of 0.45°C to 0.79°C (p < 0.001, 95% CI: 0.29–0.39°C) at 1.5T, and from 1.25°C to 1.44°C (p < 0.001, 95% CI: 0.13–0.25°C) at 3T (Fig 6). When this comparison was repeated for the GE-EPI sequences at 1.5T, differences were not significant (p = 0.652).

### Effect of stimulation setting on electrode heating

We did not observe an effect of IPG stimulation setting (‘On’ vs. ‘off’ stimulation; p = 0.42) upon **Δ**T at 1.5T during TSE sequences. However at 3T, **Δ**T was greater when the stimulator was switched off, increasing from a mean of 1.24°C to 1.44°C (p <0.001, 95% CI: 0.14–0.26°C) (Fig 6).

For the GE-EPI sequence at 1.5T, active stimulation increased electrode **Δ**T from a mean of 0.43 to 0.51°C (p = 0.0072, 95% CI: 0.02–0.14°C), an increase of 0.08°C, which is comparable with the measurement precision of our thermometer (±0.1°C).

### Effect of position within the body-coil on electrode heating

**Fig 7:**
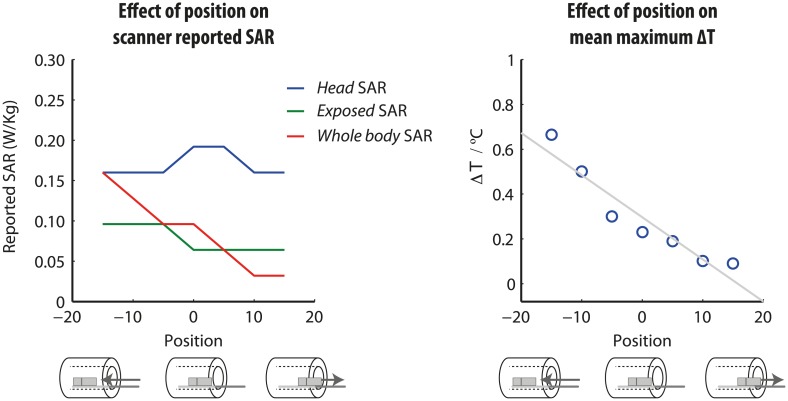
The effect of phantom position relative to the body-transmit coil on electrode-tip temperature increase. In contrast to the head-transmit coil, which moved with the patient table, the body-transmit coil is embedded at a fixed position in the scanner bore. By displacing the patient table we were able to systematically alter the position of the DBS circuit with respect to the body-transmit coil, as illustrated beneath the x-axis.

Test-object position within the body coil impacted significantly on electrode heating for the same MRI acquisition sequence and parameters. Linear regression revealed a significant effect of position (beta = -0.02, T = -6.87, p <0.001); the further the phantom was moved *into* the scanner, the greater the electrode tip ΔT. The highest **Δ**T (0.9°C) occurred when the phantom was displaced 15cm *into* the scanner (Fig 7). Scanner-reported SAR values varied with phantom position and are presented for completeness (Fig 7). *Head SAR* was a maximum (0.2 W/Kg) at 0 displacement, but reduced to 0.16 W/Kg at larger displacements in either direction. Both scanner-reported *body SAR* (max = 0.16 W/Kg, min = 0.03 W/Kg) and scanner-reported *exposed SAR* (max = 0.10 W/Kg, min = 0.06 W/Kg) reduced as the displacement out of the bore (i.e. +ve displacement) increased.

### Effect of fMRI sequences on IPG output at 1.5T

**Fig 8:**
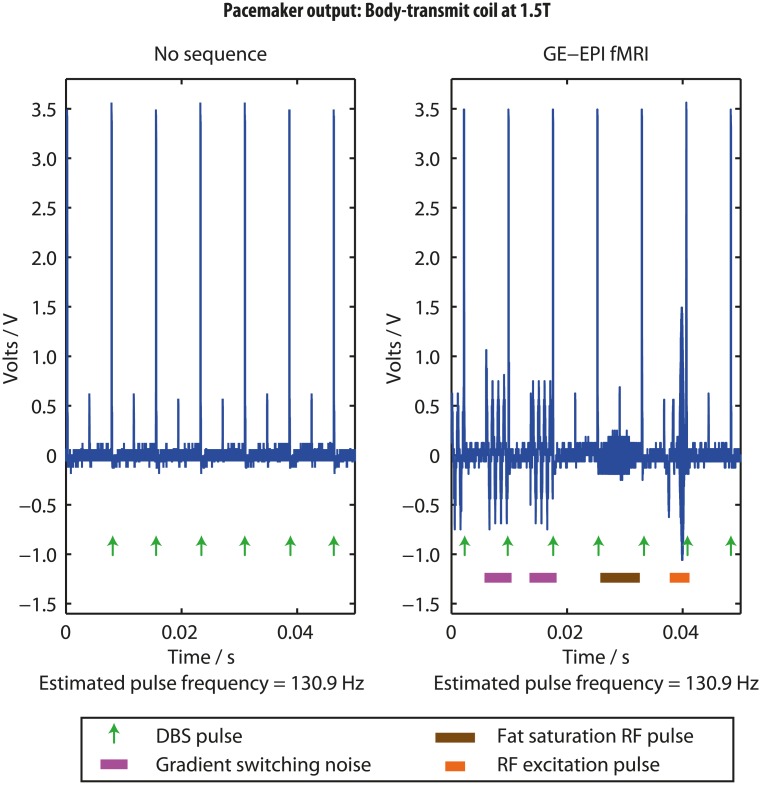
The implantable pulse generator voltage output during a typical GE-EPI fMRI sequence.

The IPG output voltage waveform recorded outside of the scanner exhibited regular discharges with frequency and amplitudes matching the programmed settings (estimated to be 130.9Hz), confirming our recording circuit functioned as expected. Fig 8 shows representative plots of the voltage between one active electrode contact and the IPG body captured during scanning using the body-transmit coil. As previously observed with head-coil transmission [27], GE-EPI sequences produced high frequency (exceeding the oscilloscope Nyquist sampling frequency) signals due to the sequence RF pulses, and lower frequency signals (~1000 Hz) arising from the switching magnetic field gradients. The RF excitation pulse induced a large amplitude signal (peak amplitude <2V), whereas the fat saturation RF pulse produced a lower amplitude (<0.5V) component. The IPG pulses with unchanged amplitude and frequency appeared superimposed upon and independent of the MRI-induced signals components. Similar results (data not presented) were obtained using the head-transmit coil.

### Effect of lead immersion in phantom gel

Routing the DBS leads along the phantom *outer wall* rather than through the conducting gel produced similar maximum **Δ**T to those obtained in the earlier experiments, and were consistent between the two repeat sessions. **Δ**T at the electrode tips with the leads immersed were <0.2°C lower than when the leads were externally routed (Table 1).

**Table 1:** The effect of lead immersion in the phantom gel.

	Maximum ΔT at electrode tips (°C)			
	Leads routed entirely in gel		Leads routed along phantom external wall	
	Left electrode	Right electrode	Left electrode	Right electrode
Session 1	0.25	0.28	0.40	0.30
Session 2	0.33	0.19	0.55	0.24

## Discussion

This series of experiments provides evidence that cranial MRI at 1.5T using the body-transmit coil in patients with new DBS systems can be collected without harm to the patient, provided a restricted SAR threshold is adopted. We now discuss how these findings relate to current safety data on implanted DBS devices, and their implications for potential confounding of fMRI data.

### The safety of active DBS during fMRI using the body-transmit coil

#### Guidelines

Current UK and international guidelines specify that MRI-induced heating should not cause cerebral temperatures to exceed 38°C, requiring any intra-cerebral heating at the electrodes to be ≤1°C [43]. Our results suggest that this safety specification can be achieved during 1.5T cranial MRI with a Medtronic ActivaPC system, regardless of transmit coil used, providing the head SAR is limited to ≤0.2 W/Kg. This SAR limit exceeds that currently recommended by the device manufacturer [44] and it is important to state that MRI of patients with implanted hardware outside of the manufacturer’s instructions should only be performed following a comprehensive local risk assessment specific to the MRI system and particular DBS hardware under supervision from MRI experts.

#### Experimental design

MRI-induced heating was measured in the vicinity of the electrode tips, as these regions are in direct contact with neural tissue *in vivo*, and have previously been identified as those showing the greatest MRI-induced ΔT [26,27,30,38,40,45–47]. Heat dissipation in these regions occurs primarily via thermal conduction and convection within the gel; it should be noted that, despite similar thermal properties, the *in vitro* gel measurements conservatively over-estimate MRI-induced heating obtained *in vivo*, where heating is presumably reduced by cerebral blood flow [48,49].

TSE sequences with a relatively high SAR were chosen deliberately so as to generate **Δ**T sufficient for accurate measurement given the sensitivity of the thermometry system. Heating was consistently largest at the electrode tips, with the left electrode displaying the greatest **Δ**T. Such asymmetry has been reported elsewhere and is believed to be due to asymmetry in the DBS circuit with respect to scanner field orientation [27,47,50], combined with asymmetries in the scanner B1 transmit field within the phantom due permittivity boundary conditions. It was unlikely that local gel property variation caused this asymmetry, as this was observed consistently throughout all measurements, including separate temperature recordings and multiple gel preparations.

#### Effect of transmit coil at 1.5T

Using the head-transmit coil at 1.5T, the average maximum ΔT was 0.45°C, compared to 0.79°C using the body-transmit coil. With the electrode tips positioned at the scanner isocentre, scanner-calculated head and body SAR remained constant (0.2 and 0.1 W/Kg, respectively) for both coil arrangements, suggesting ΔT differences were not due to varying RF pulse power calibration. The different ΔT are most likely explicable by differences in the area of the DBS circuit exposed to the RF B_1_-field in each case, changing the conditions for electrical coupling according to Maxwell’s equations [26]. A similar mechanism presumably underlies our subsequent finding that ΔT is also dependent upon position of the phantom within the body-transmit coil. Furthermore, differences in the design of the RF coils (and hence the associated electric fields) may also play a role.

#### Position in the scanner during body-transmit MRI at 1.5T

It is important to consider the safety of cranial MRI under the plausible circumstance of a patient poorly positioned in the scanner. Heating produced by body-coil transmit MRI at 1.5T was significantly predicted by the position of the phantom in the scanner. ΔT increased the further into the scanner the phantom was moved, consistent with more of the DBS circuit being exposed to the RF field, thus increasing the induced current magnitudes. The pulse sequence parameters were held constant between positions; interestingly, the scanner-reported *head* SAR did not directly explain the temperature change. Rather, in a separate regression, the *whole body* SAR significantly predicted the observed ΔT (P <0.05). This suggests that the *whole body* SAR is a better means of confirming safety when using the body-transmit coil, and may provide a more meaningful metric for defining maximum safe RF exposure. Analogous experiments using the head-transmit coil were not necessary, since in this case the position of the patient (and hence DBS circuit) relative to the coil is essentially fixed for all examinations. Scanning with the patient displaced +150mm from isocentre would be effectively result in their head being almost outside the scanner. This would be a rather extreme case of patient misplacement, but the corresponding data were however collected for completeness.

#### 3T MRI using our positive control SAR produced heating >1°C

Scanning at 3T with our positive control medium SAR sequence produced ΔT exceeding 1°C suggesting an increased thermal risk compared with similar acquisitions at 1.5T. Although relatively modest when using the head-transmit/receive coil (<2°C), 3T heating did in this case exceed the HPA 2008 recommendations. Since there is limited published evidence regarding safe temperature thresholds in this context, we have chosen in our practice to adhere to these stringent recommendations and avoid 3T scanning until more information is available. It should be noted that some published imaging studies in both human patients and animal models of DBS have been more liberal in this respect [51–54], with no reported deleterious effects. It should also be noted that we only tested our positive control TSE sequence at 3T, not the lower-SAR GE-EPI sequences that are more likely to be required in research protocols.

#### Pre-scan heating

The instance of 10°C heating observed during the pre-scan procedure for our positive control TSE sequence at 3T with the body-transmit coil is a cause for concern and further investigations are required to determine the precise origins of this unexpected ΔT. In any case this observation highlights the importance of considering the effects of RF deposition during the pre-scan phase of MRI acquisitions as well as during play-out of the main imaging sequence. Throughout the data collection process, magnitudes of pre-scan heating at 1.5T remained in keeping with those associated with the TSE sequence.

#### The safety of induced electrical components in the DBS circuit

The oscilloscope recordings faithfully demonstrated the expected qualitative characteristics of the DBS equipment output, and induced signals characteristic of the MRI scanner operation. The MRI sequences induced intermittent RF and low-frequency gradient-switching signals in the DBS circuit, consistent with previous reports [26–28]. The safety of induced voltages is dependent on both their frequency and amplitude. The signal frequency determines the risk of depolarisation and direct neural stimulation. The RF frequencies produced by these MRI systems exceed 60MHz, too high to cause neuronal stimulation. The induced signals arising from gradient-switching are significantly lower in frequency (≈1 kHz), but above any stimulation frequencies known to have therapeutic effect. The induced voltage peak-to-peak amplitudes were in all cases less than 1.5V, i.e. approximately half of the therapeutic DBS pulse amplitude, and therefore presumed to be safe.

### Potential MRI-DBS interaction confounds to fMRI in DBS patients

#### IPG delivers programmed DBS during fMRI

Typical fMRI sequences did not produce any changes to DBS frequency, pulse width or amplitude, or general function. Previous studies with older DBS systems have reported spontaneous switching on/off of the IPG [19]. Carmichael et al. reported that, for a different DBS device, approximately 10% of DBS pulses following a 90° RF pulse had extended inter-pulse intervals. This was not detected using the ActivaPC system, which may reflect advances in IPG design. As discussed above, the MRI-induced low frequency signals observed are outside the frequency range of therapeutic DBS. Furthermore, they appeared during both ‘ON’ and ‘OFF’ stimulation conditions suggesting they do not confound any comparisons of the BOLD response between conditions.

#### Temperature should not confound ON vs. OFF comparisons

Any MRI-related local temperature-related physiological or other signal differences due to ON versus OFF stimulation states could complicate an fMRI study comparing the two conditions. During the TSE sequences ΔT differences between ON and OFF were not significant at 1.5T, although a significant effect was detected during GE-EPI at 1.5T (active DBS increasing the temperature by an average of 0.08°C). However, this small difference is comparable with the sensitivity of our thermometry system and we conclude that any effect is of negligible practical importance. A previous investigation at 3T also reported a dependence of ΔT upon DBS state, where activating the DBS increased mean heating by 0.08°C, although formal comparisons were not presented [51]. One can speculate that if the effect is indeed true, it may be due to differences in the circuit impedances between the two stimulation conditions.

### Limitations

There are a number of important caveats to our findings, centring on their generalizability to other settings.

Firstly, electrode heating is dependent on the specific geometry of the exposed circuit; we report results from a specific arrangement simulating a (single) IPG situated in the pectoral region, exposed to cranial MRI. More extensive investigations are required to confirm the safety of scanning patients with abdominal and or multiple IPGs, or for MRI targeting other body regions (e.g. spine, abdomen).

Furthermore, the phantom was intended to model a typical DBS arrangement for patients that have been operated at our centre. Different geometric orientations of the DBS components, lead coiling configurations, and indeed the direction of lead coiling may impact on electrode heating. It has been noted that lead coiling variations influence the MRI artefact pattern surrounding the electrode tip [39], suggesting there may be a small resulting RF field perturbation. More detailed future measurements may define more precisely the magnitude of heating variations caused by spare-lead coil orientation and polarity, although practical clinical experience suggests that under appropriately controlled MRI conditions this does not impact significantly on safety with the lead arrangements commonly used.

Our measurements comparing electrode tip **Δ**T with the lead extensions immersed in the tissue-simulating gel with **Δ**T obtained with the lead extensions external to the phantom wall revealed consistent but small differences. For the specific configurations tested, these differences were too small to influence our conclusions regarding safe MRI conditions for these devices, suggesting small variations in *in vitro* model configurations are acceptable from the standpoint of defining safe limits incorporating conservative added error margins. More extensive tests, or software simulations, may be required to determine the influence of lead routes upon safety conclusions under different circumstances where the measured **Δ**T for the specified SAR are close to safe *in vivo* limits.

Like any model, this *in vitro* model has a number of limitations. For this reason, we have adopted a very conservative safety threshold. While models employing cadavers and animals could add extra insight, they are not without their own limitations (e.g. anatomical and lead geometry discrepancies, and post mortem changes of thermal and electrical tissue properties) and are beyond the scope of this paper. Gel phantoms have been used routinely in similar studies and their use is recommended by the ASTM technical standards and ISO technical specification.

Importantly, these results are based upon scans limited to a scanner-reported head SAR≤0.2W/Kg in all cases, as obtained on our specific MRI systems. While the use of head SAR to predict electrode heating has been previously explored [31], it is important to note that the models used to calculate scanner-reported SARs are highly system dependent, varying between manufacturer and specific MRI models, and thus may vary between centres [46]. Also the SAR values reported by the MR system are not intended to estimate actual values that would be present in a patient with an implant or device, which in test objects may be may be determined by calorimetry [55].

Therefore, while our results provide strong experimental evidence that MRI data acquisition may be safely performed in DBS patients with this equipment, far more extensive testing is required for the generalisation of specific safety thresholds to other centres using different DBS or MRI equipment. Until more general guidance from the equipment manufacturers is available, it therefore remains important to perform local risk assessments and *in vitro* measurements to confirm safety before undertaking procedures that do not conform with device manufacturers’ instructions-for-use.

Finally, while this work addressed minimising tissue-heating by limiting the overall sequence SAR, the growing availability of multi-channel RF excitation technology could in the future offer alternative efficient methods of obtaining high quality images whilst controlling local RF power deposition to safe levels [56].

We conclude that with suitable precautions, cranial MRI of patients with fully implanted DBS systems, both active and inactive, can be safely performed using the body-transmit coil at 1.5T, potentially allowing higher quality clinical and research acquisitions in these patients. *In vitro* thermometry suggests that while electrode heating remains the principle safety concern, this can be controlled to safe levels under a strictly defined acquisition protocol. The DBS systems functioned as programmed during typical GE-EPI fMRI sequences using the body-transmit coil, and the MRI-induced voltages are not such as to pose a risk to patients, or confound fMRI study designs. Importantly, although our results are very encouraging for the prospects of safe DBS-MRI at 1.5T, we highlight additional concerns regarding prescan-related MRI safety in these patients at 3T, which warrant further investigation.

## Supporting Information

S1 Fig The effect of ‘gel age’ on TSE-induced heating. As a representative assessment of measurement reproducibility and gel stability, we compared the heating produced by our TSE sequence when the gel was freshly prepared on the day of scanning, and when the gel was 8 days old. Our results demonstrate that at 8 days, the heating trend throughout the scan remains qualitatively similar, with no significant difference identified in maximum heating (p = 0.13).
(TIF)
