# Supplementary figures and images for: The Safety of Using Body-Transmit MRI in Patients with Implanted Deep Brain Stimulation Devices
Source: PLoS One. 2015 Jun 10;10(6):e0129077. doi: 10.1371/journal.pone.0129077 (PMC4465697; doi:10.1371/journal.pone.0129077)

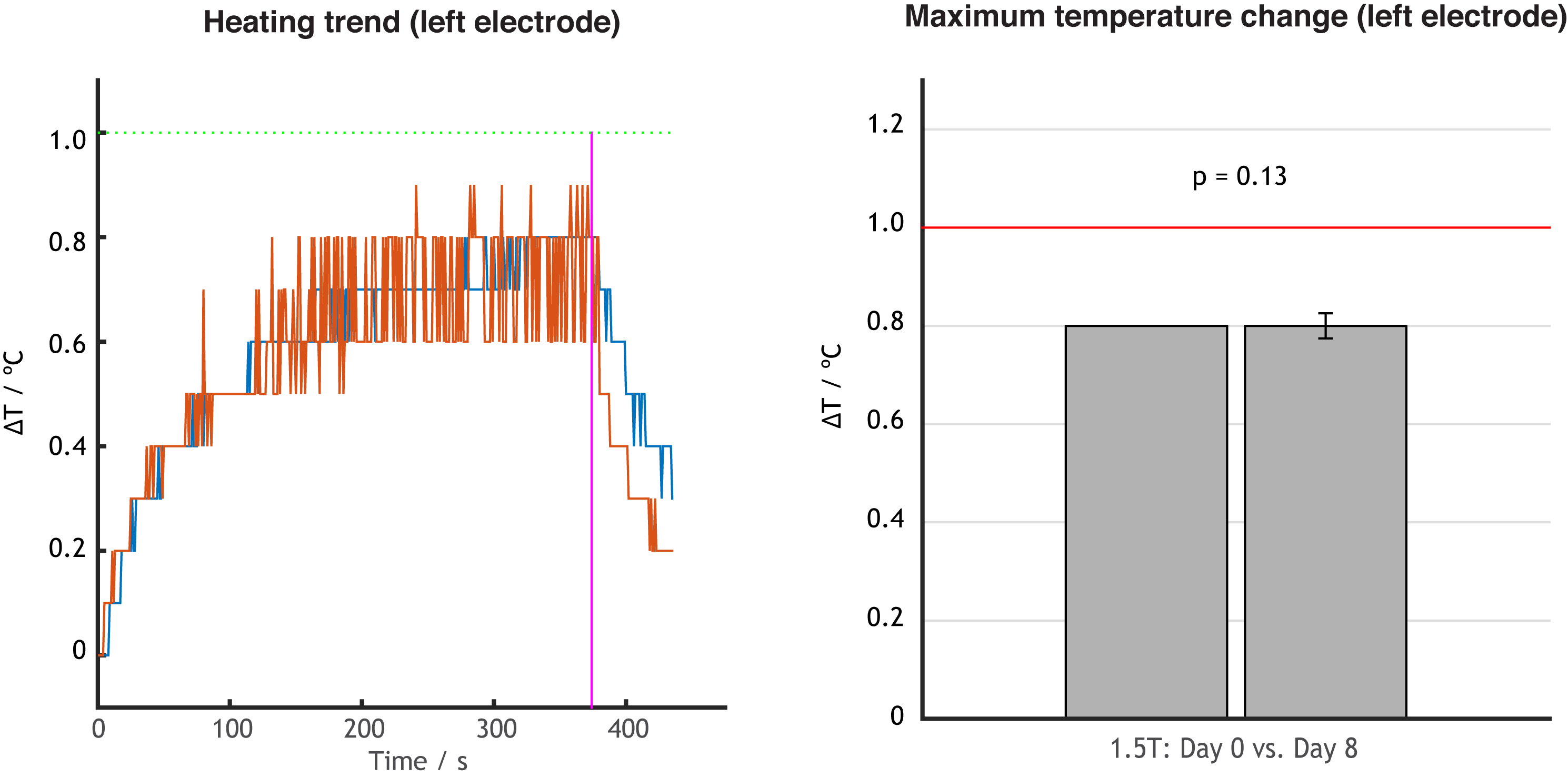

Supplement: S1 Fig — As a representative assessment of measurement reproducibility and gel stability, we compared the heating produced by our TSE sequence when the gel was freshly prepared on the day of scanning, and when the gel was 8 days old. Our results demonstrate that at 8 days, the heating trend throughout the scan remains qualitatively similar, with no significant difference identified in maximum heating (p = 0.13). (TIF) [file pone.0129077.s001.tif]
